# Longitudinal Patterns of Food Procurement Over the Course of the COVID-19 Pandemic: Findings From a Canadian Online Household Survey

**DOI:** 10.3389/fpubh.2021.752204

**Published:** 2022-01-20

**Authors:** Daiva E. Nielsen, Katherine Labonté, Irem Karamanoglu, Hannah Yang Han, Mandana Tavanaei, Paul-Guy Duhamel, Luis B. Agellon, Catherine Paquet, Laurette Dube

**Affiliations:** ^1^School of Human Nutrition, McGill University, Montreal, QC, Canada; ^2^Faculté des Sciences de l'administration, Laval University, Quebec, QC, Canada; ^3^Desautels Faculty of Management, McGill University, Montreal, QC, Canada

**Keywords:** food procurement, online grocery, risk perceptions, COVID-19 pandemic, online survey

## Abstract

**Introduction:**

Consumer food procurement during the COVID-19 pandemic has been understudied. This investigation aimed to longitudinally evaluate food procurement patterns, concern of virus exposure in grocery retailers, and food access challenges over the pandemic among a sample of households in Quebec, Canada.

**Methods:**

Online surveys were collected at three time points of the pandemic: first wave in spring 2020 (lockdown period), summer 2020 (deconfinement period), and second wave in winter 2021 (curfew period). Respondents were the household's primary grocery shopper (*n* = 491). Non-parametric tests and multivariable logistic regression were conducted to compare responses over time and to evaluate characteristics of respondents who regularly used no-contact grocery methods (store pick-up or home delivery).

**Results:**

Frequency of in-store grocery shopping was lowest during the lockdown (once per week or less), and significantly increased over time to resemble pre-pandemic frequency. Concern of virus exposure in grocery retailers and disinfection/discarding of food packaging was highest during the lockdown, but significantly decreased over time. At all time points, use of public transit, walking or cycling for grocery shopping was associated with regular use of no-contact grocery methods (curfew odds ratio (OR): 3.13 (95% confidence interval 1.60, 6.14). Age (60 years+) was associated with regular use during the lockdown [OR: 2.27 (1.13, 4.59)].

**Conclusion:**

Among our sample, frequency of in-store grocery shopping was lowest and concern of virus exposure in stores was highest during the lockdown period. No-contact grocery use was associated with transportation mode and potentially with personal risk perception (age).

## Introduction

The COVID-19 pandemic has had profound societal impacts that have disrupted activities of everyday life, such as food procurement. Lockdowns, loss of income, and disruptions in food supply chains have impacted consumer food access and food security globally, with severe ramifications amongst the world's poorest (1). The World Food Program (WFP) estimates that the number of people experiencing food crises due to the pandemic could double around the world if appropriate action is not taken (2); however, the pandemic's impact on global food security is considered to stem from issues related to consumer food access rather than food availability (3). Therefore, investigations into ways in which individuals organized themselves around the basic act of procuring food over the course of the pandemic are important to inform strategies for food retailers and consumers in the event of future public health emergencies. India, South Africa, and the United Kingdom were observed to have had sharp decreases in in-person grocery shopping during the beginning of the pandemic, potentially a result of these countries' strict and sudden lockdown implementations (4), and the global use of no-contact grocery methods (ordering online or by phone) have increased dramatically during the pandemic (5–7). Despite the rapid increase in no-contact grocery methods including store pick-up and home delivery, few investigations have evaluated consumer experiences with these methods and other outcomes related to food procurement. Therefore, this investigation used online surveys to longitudinally examine household grocery shopping frequency and method (in-store vs. no-contact), concerns over virus exposure in grocery stores and through grocery products, food access challenges, and indicators of food insecurity over the course of the COVID-19 pandemic among a convenience sample of households in the province of Quebec, Canada.

Quebec is the largest Canadian province by area and second largest by population with 8,164,361 residents (2016 Census) (8). During the first wave of the pandemic, Quebec had the highest numbers of COVID-19 cases in Canada and a provincial lockdown period was in effect during spring 2020 where non-essential services were closed. Face mask use became mandatory in all indoor public spaces, including food retailers, in summer 2020 (9). COVID-19 prevalence fell that summer only to rise again during fall 2020 and through winter 2021 (second wave) (10). To control the spread of the virus during the second wave, the province instituted a curfew that required individuals to be at their home (or in a small perimeter surrounding their home for dog walking) between the hours of 8 pm−5 am in the areas with highest COVID-19 prevalence (9:30 pm−5 am in areas with lower prevalence). Retailers including grocery stores closed at 7:30 pm, but delivery of take-out food was permitted during curfew hours. Our data collection occurred between spring 2020—winter 2021, capturing the lockdown period of the first wave, deconfinement period, and curfew period of the second wave. A third wave occurred in spring 2021, but this investigation had been completed prior to its onset.

We previously reported results from our first survey conducted during the lockdown where we observed a reduction in the frequency of in-store grocery shopping and an increase in the use of online grocery shopping compared to before the pandemic (11). Concern of virus exposure in grocery stores and disinfecting/discarding food product packaging were also prevalent. This present investigation reports on changes to these outcomes that were re-evaluated at two additional time points: summer 2020 when restrictions eased (deconfinement period) and during the second wave in winter 2021 (curfew period). The primary outcomes of interest were changes in the frequency of grocery shopping (in-store and no-contact) and characteristics of regular users of no-contact grocery methods. We evaluated the following two hypotheses: (1) In-store grocery shopping frequency would be lowest at the lockdown period and return to pre-pandemic patterns by the end of the investigation, reflecting consumer adaptation to the public health situation over time; and (2) Sociodemographic characteristics of regular users of no-contact grocery methods would be stable across time points, reflecting consumer preference for this grocery method (12). Other outcomes of interest included changes in concern of virus exposure in grocery stores and from grocery products, methods of meal preparation, and food access challenges (including during self-isolation). These were considered exploratory analyses given characteristics of our sample and lack of existing literature upon which to form hypotheses for each outcome. Findings from this research are anticipated to assist in informing retail and public health considerations around food access in the event of future public health emergencies.

## Methods

### Survey Overview and Timeline

An open online household survey investigation with three time points over ~1-year was conducted *via* the platform SurveyMonkey. Recruitment was facilitated prior to the first survey through a radio broadcast, digital advertising in online media outlets, a social media campaign, and through professional networks. Informed consent was collected at the time of participation in the first survey, where respondents could also provide consent to be contacted for the follow-up surveys. Those who provided consent for recontact were e-mailed when the follow-up surveys were available for completion. Two e-mail attempts were made for the second survey and three attempts for the final survey, ~1 week apart, after which respondents were considered lost to follow-up. An incentive was added to the final survey to attempt to retain participation (random draw for a $20 electronic gift card to participating local retailers), but no incentive was provided for the first or second survey. The survey respondent was the household member who was primarily responsible for grocery shopping. Each survey required 15–20 mins to complete.

Responses to the first survey (lockdown) were collected between May 20—June 4, 2020. This survey retrospectively probed for information beginning from March 13, 2020 (the start of the lockdown) and some items enquired about 2019 (methods of meal preparation, household food situation). Respondent postal codes were collected and linked to the Statistics Canada Postal Code Conversion File (August 2018 release) to determine region of residence and assign urban and rural classifications. The second survey (deconfinement) was collected between August 15–30, 2020, capturing information when the province had re-opened and the prevalence of COVID-19 was low. The third and final survey (curfew) was collected between February 15—March 14, 2021, during the second wave of the pandemic when the provincial curfew was in effect. A total of 1,955, 658, and 621 individuals completed the lockdown, deconfinement, and curfew surveys, respectively. Analyses for the present investigation were conducted on the sample of users that completed all three surveys to ensure that comparisons were made on the same group of individuals.

### Survey Development

The longitudinal surveys consisted of between 16 and 19 items, depending on the number of follow-up questions that applied, most of which were repeated at each time point (survey questions are presented in the Supplementary Tables 1–3, as well as in respective Tables/Figures). In addition, eight sociodemographic questions that were collected on the first survey (lockdown) were considered in the present analyses. The surveys were developed by the research team, comprised of investigators from nutrition science and consumer science, and were available in English and French. Face validity and pilot testing were conducted with a pilot sample of eight community-dwelling participants to correct any leading questions or unclear wording. French translations were back translated to English and tested among bilingual (*n* = 4) and francophone (*n* = 4) individuals to ensure comprehension in mother tongues. Responses from the first survey were analyzed and assessed to inform decisions for any modifications to survey items (addition/removal of questions) for the follow-up surveys.

### Study Outcomes

This investigation evaluated four themes: grocery shopping frequency and methods, concern of virus exposure and mitigation behaviors, methods of meal preparation, and food access challenges (including during 14-day self-isolation). The first theme contained our primary outcomes of interest. Frequency of in-store grocery shopping and frequency of using no-contact grocery methods were assessed at all time points using a 7-point scale: daily or more, 4–6 times per week, 2–3 times per week, once per week, 1–3 times per month, less than once per month, or never. Completeness of no-contact grocery methods was assessed at all time points as a proxy for reliability of the service, using a 4-point scale evaluating whether the grocery order contained: everything that was ordered, almost everything, some products not included, or many products not included. The wait time for no-contact grocery orders to arrive was also evaluated at all time points using a 4-point scale: 1–3 days, 4–7 days, 8–13 days, or 2 weeks or more. We evaluated the sociodemographic characteristics of regular users of no-contact grocery methods, defined as frequency of use of at least once per week, using sociodemographic data from the lockdown (baseline) time point and no-contact grocery use responses from all time points.

The remaining themes were evaluated in exploratory analyses and included concern of virus exposure in grocery stores and mitigation strategies, methods of meal preparation, and food access challenges (including during self-isolation). Readers are directed to Supplementary Table 2 for the list of specific questions and response options for these themes, beginning from item 6. Note that the lockdown survey items regarding food product availability were not repeated on the deconfinement survey due to improvement of the public health situation leading to easing of restrictions. Thus, it was considered unlikely that food products would be less available at that time and the questions were removed to reduce survey completion time. The items were included on the curfew survey when COVID-19 prevalence had increased, and public health restrictions tightened. Two and four items from the Household Food Security Survey Module were included on the lockdown and follow-up surveys, respectively, to evaluate indicators of food insecurity (13). All time points included an item on household food situation (item 12) and an adapted question on skipping meals/reducing food intake as a form of food rationing (item 13 a and b), a coping strategy to conserve one's food supply that is a potential indicator of vulnerability to food insecurity (14). Food rationing behavior was defined as any of the following reasons for reducing food intake: food not lasting between grocery trips, unable to afford to buy more food, or saving food for other household members. Results from the lockdown survey revealed common reports of skipping meals for non-income related reasons (such as loss of appetite due to stress, or health consciousness). Therefore, two income-related food insecurity items were added to the follow-up surveys to specifically evaluate income-related vulnerability (items 14–15). The present investigation was not designed to assess the prevalence of food insecurity, but these questions were used to evaluate the potential for vulnerability to food insecurity over the course of the investigation. Ethics approval was obtained from the McGill University Faculty of Agriculture and Environmental Sciences Research Ethics Board (#20-05-021). As per ethics requirements, all survey items were optional to respond to. Thus, minor variations in sample sizes across questions occur due to non-response.

### Statistical Analyses

Statistical analyses were conducted with SPSS version 24 and all p-values were two-sided with alpha level of 0.05. Descriptive statistics were calculated to obtain frequencies (%) of responses for each item. Changes in repeated survey items were analyzed using non-parametric tests. Specifically, categorical data that were binary in nature and repeated on all three surveys were analyzed using Cochran's *Q*-test to determine whether an overall significant difference across time points was evident. Significant results were further assessed with the McNemar test to evaluate comparisons of time point pairings (lockdown vs. deconfinement, and deconfinement vs. curfew). The Friedman test was used to compare responses to ordinal items from all three time points to determine whether an overall significant difference was evident. Similarly, significant results were further assessed with the Wilcoxon signed-rank test to evaluate comparisons of the same above-described time point pairings. Some multiple-choice survey items included an “Other (please specify)” free text response option. These free text responses were analyzed using content analysis with two researchers involved in coding and content extrapolation using an inductive approach (15). MT read free text responses several times and developed a codebook, which DEN reviewed. MT assigned codes and suggested content themes, which DEN reviewed. Discordant themes were discussed and 100% consensus was achieved.

Multivariable binary logistic regression was conducted to identify sociodemographic characteristics of respondents who regularly used no-contact grocery methods at each study time point (yes/no dependent variable). Adjusted odds ratios were examined from a single model for each time point that included the following socio-demographic factors as independent variables: age [under 60 (reference), 60 years and older] [this cut-off was evaluated because being 60 years of age or older is a risk factor for severe illness from COVID-19 (16)], gender [female (reference), male], marital status [married (reference), single, divorced/separated/widowed], total household income [under $50,000 (reference), $50,000-$100,000, over $100,000], children under the age of 18 years residing in the household [no (reference), yes], household size [single individual (reference), 2 individuals, 3 or more individuals], primary mode of transportation when grocery shopping [car (reference), public transit/walking/cycling], baseline concern over the COVID-19 pandemic [low (reference), medium, high], and urban/rural region of residence [large population center (reference), small and medium population center, rural].

## Results

A total of 491 respondents completed all three surveys (47% of those who consented to be contacted for the follow-up surveys). Response rates for each survey item were high, with the lowest rate being 97%. The respondents resided in 15 of the 18 administrative health regions of the province, with approximately half residing in Montreal (Supplementary Material). Table 1 displays characteristics of these respondents. At baseline, the majority of respondents were moderately to extremely concerned about the pandemic (88%). Most respondents resided in urban areas with a mean ± standard deviation household size of 2.6 ± 1.4, that is similar to the average household size in Quebec (2.3 individuals) (8). The most common response for total household income among our sample ($50,000–$99,999) aligned with the median total household income of Quebec economic families ($79,378 from Census 2016), defined in the Canadian Census as a group of two or more persons who live in the same dwelling and are related to each other by blood, marriage, common-law union, adoption or a foster relationship (8). Thus, the present sample appears similar in demographic composition to Quebec families living in urban regions. Characteristics of this sample of respondents align with those of the full set of the lockdown (baseline) survey respondents (11). Supplementary Table 4 presents the distribution of respondents across the province's 18 administrative health regions.

**Table 1:** Baseline characteristics of respondents.

**Characteristic**	**n (%)^†^**
Age group (years)	
18–39	153 (31%)
40–59	216 (44%)
60 and older	122 (25%)
Total	491
Gender	
Female	445 (91%)
Male	44 (9%)
Preferred to specify	2 (<1%)
Total	491
Language	
French	271 (55%)
Total	491
Total household income	
< $20,000	18 (4%)
$20,000–$49,999	69 (14%)
$50,000–$99,999	190 (39%)
$100,000–$149,999	119 (25%)
$150,000–$199,999	52 (11%)
≥$200,000	36 (7%)
Total	484
Marital status	
Never married	92 (19%)
Married/Common-law	334 (68%)
Separated/Divorced	53 (11%)
Widowed	10 (2%)
Total	489
Urban vs. rural residence	
Large population center	369 (78%)
Medium population center	27 (6%)
Small population center	28 (6%)
Rural	48 (10%)
Total	472
Household size	
Single individual	109 (23%)
2	179 (37%)
3 or more	195 (40%)
Mean ± standard deviation	2.6 ± 1.4
Total	483
Mode of transportation for grocery shopping	
Car	381 (78%)
Public transit, walking, cycling	109 (22%)
Total	490
Baseline concern over pandemic	
Low (Not at all + Slightly concerned)	61 (12%)
Medium (Moderately concerned)	162 (33%)
High (Very + Extremely concerned)	268 (55%)
Total	491

† *Percentages may not total to 100% due to rounding*.

### Grocery Patterns and Use of No-Contact Methods

**Figure 1:**
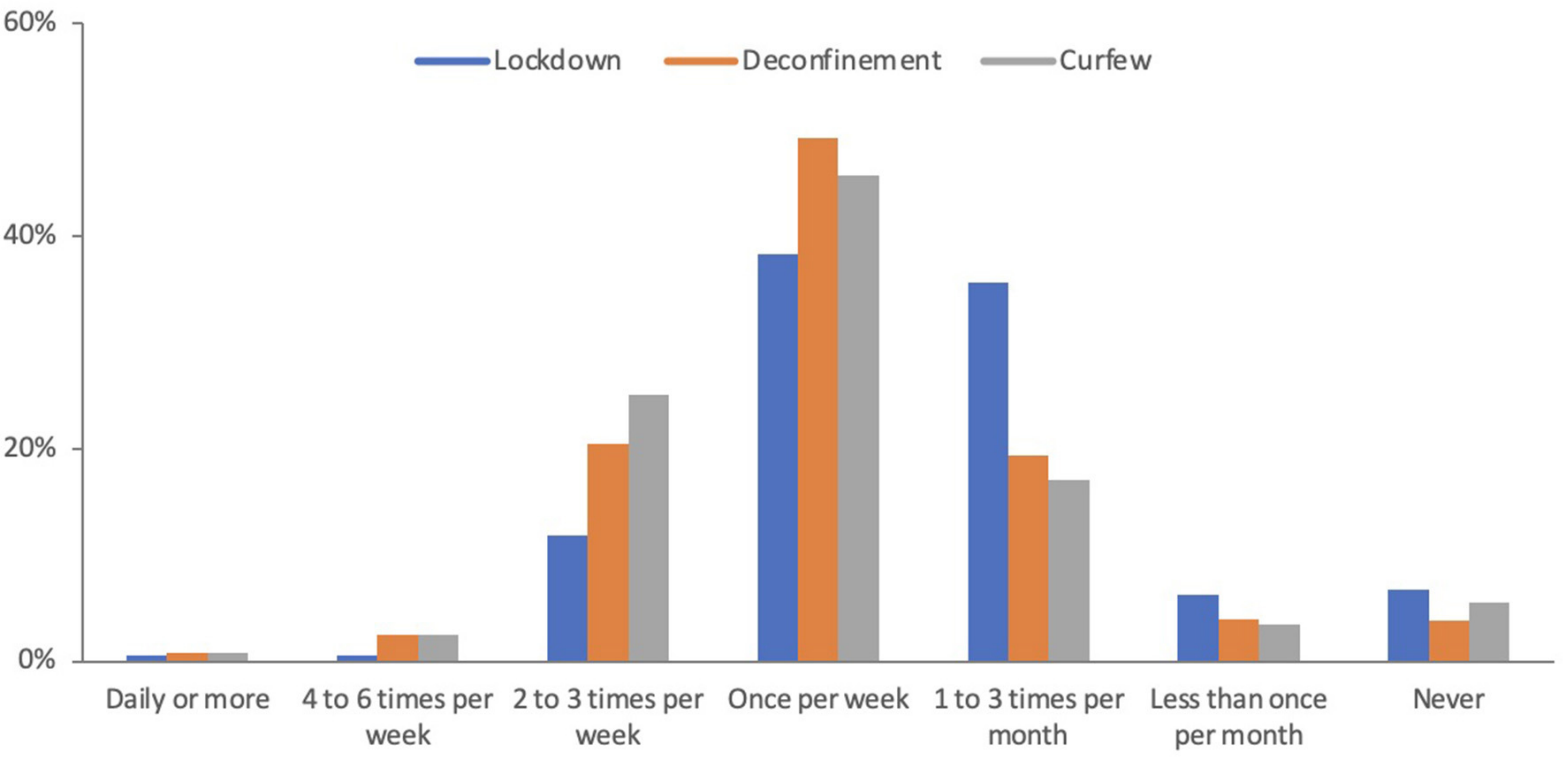
Grocery shopping frequency over the COVID-19 pandemic. “Overall, between *(time period)*, how often did you/your household delegate physically go into astore to shop for groceries?” Wilcoxon signed-rank test: Lockdown vs. Deconfinement *p* < 0.001, Deconfinement vs. Curfew *p* = 0.537.

**Figure 2:**
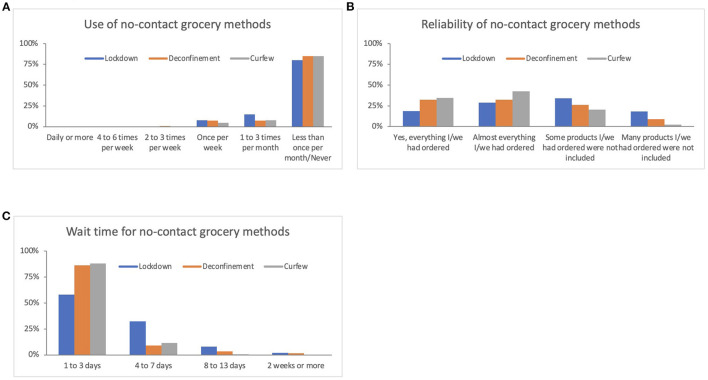
Changes in use, completeness, and wait time for no-contact grocery methods. **(A)** “Between *(time period)* how often did your household utilize grocery pick-up or home delivery?” Wilcoxon signed-rank test: Lockdown vs. Deconfinement *p* < 0.001, Deconfinement vs. Curfew *p* = 0.318. **(B)** “If you used grocery pick-up or delivery, were all the products your household ordered included in what you received?” Wilcoxon signed-rank test: Lockdown vs. Deconfinement *p* < 0.001, Deconfinement vs. Curfew *p* = 0.005. **(C)** “If you used grocery pick-up or delivery, on average, after placing your order how long did you have to wait to receive your groceries?” Wilcoxon signed rank-test: Lockdown vs. Deconfinement *p* < 0.001, Deconfinement vs. Curfew *p* = 0.260.

Figure 1 presents patterns of in-store grocery shopping frequency across time points. During the lockdown period in the first wave of the pandemic, most respondents selected that they went in-store grocery shopping either once per week (38%) or one to three times per month (36%). Some respondents (11%) reported never shopping for groceries in-store during the lockdown period. The frequency of grocery shopping significantly changed across the time points (Friedman test *p* < 0.001). Specifically, the frequency increased at the deconfinement time point and remained similar at the curfew time point, where most respondents indicated that they went in-store shopping once per week (46%) or 2–3 times per week (25%). At all time points, most respondents (>50%) indicated that only one member of the household was responsible for grocery shopping (Supplementary Table 5). However, the proportion of respondents who reported that grocery shopping was done by more than one member of the household increased and the proportion who reported never going in-store grocery shopping decreased after the lockdown period (in both cases, the change remained stable between deconfinement and curfew) (Supplementary Table 5). Relatedly, the frequency of no-contact grocery use significantly differed across the time points (Friedman test *p* < 0.001). It decreased between the lockdown period and deconfinement, and remained stable thereafter (Figure 2A). Figures 2B,C present responses for the completeness and wait time for no-contact grocery methods that significantly differed across the time points (Friedman test *p* < 0.001). During the lockdown period, slightly over half of the respondents reported that some or many items were missing when they received their orders. The proportion of respondents reporting missing items significantly decreased over time, indicating an improvement in completeness of no-contact grocery methods after the lockdown period. Similarly, during the lockdown period, roughly 40% of participants reported having to wait 4 or more days for their no-contact grocery order. Wait time significantly decreased between the lockdown and deconfinement, thereafter remaining stable with nearly 90% of respondents reporting a wait time of 3 days or less.

The most consistent characteristic of regular users of no-contact grocery methods was usual mode of transportation for grocery shopping (Table 2). Those who used public transit, walking, or cycling were significantly more likely to use no-contact methods regularly compared to those who used a car. Being aged 60 years or older, compared to under 60, was a significant predictor during the lockdown period, but not at the other time points. Higher total annual household incomes were significantly associated with regular no-contact grocery use, compared to the lowest income category, during the lockdown period only. Gender, marital status, children residing at home, household size, and baseline concern about the COVID-19 pandemic were not significant predictors of regular no-contact grocery use at any time point.

**Table 2:** Characteristics of regular users of no-contact grocery methods (*n* = 463).

**Characteristic**	**Lockdown OR (95% CI)**	**Deconfinement OR (95% CI)**	**Curfew OR (95% CI)**
Age			
Under 60 years old	Reference	Reference	Reference
60 years and older	**2.27 (1.13, 4.59)**	1.34 (0.65, 2.79)	1.91 (0.96, 3.83)
Gender			
Female	Reference	Reference	Reference
Male	0.54 (0.17, 1.66)	1.09 (0.40, 2.95)	0.78 (0.27, 2.22)
Marital status			
Married	Reference	Reference	Reference
Single	2.32 (0.90, 5.99)	0.47 (0.16, 1.38)	0.78 (0.27, 2.21)
Divorced/separated/widowed	0.95 (0.36, 2.51)	0.79 (0.30, 2.07)	0.65 (0.23, 1.80)
Total annual household income			
<$50,000	Reference	Reference	Reference
$50,000–$99,999	**3.06 (1.12, 8.35)**	1.34 (0.54, 3.29)	0.70 (0.29, 1.70)
$100,000+	**4.82 (1.65, 14.03)**	1.72 (0.65, 4.57)	1.19 (0.47, 2.98)
Children residing at home			
No	Reference	Reference	Reference
Yes	1.63 (0.85, 3.16)	1.12 (0.56, 2.27)	1.13 (0.57, 2.24)
Household size			
Single individual	Reference	Reference	Reference
2	1.99 (0.78, 5.04)	0.54 (0.21, 1.36)	2.24 (0.87, 5.79)
3 or more	2.30 (0.78, 5.04)	0.97 (0.34, 2.77)	1.56 (0.50, 4.89)
Mode of transportation for grocery shopping			
Car	Reference	Reference	Reference
Public transit, walking, cycling	**3.75 (1.92, 7.33)**	**5.10 (2.60, 10.00)**	**3.13 (1.60, 6.14)**
Baseline concern about COVID-19 pandemic			
Low	Reference	Reference	Reference
Medium	1.21 (0.43, 3.42)	2.31 (0.70, 7.64)	1.37 (0.41, 4.62)
High	1.85 (0.70, 4.88)	3.03 (0.97, 9.49)	2.96 (0.96, 9.15)
Urban/rural			
Large+Medium Urban	Reference	Reference	Reference
Small Urban	0.50 (0.11, 2.25)	0.71 (0.16, 3.18)	0.29 (0.04, 2.25)
Rural	0.75 (0.27, 2.07)	0.36 (0.08, 1.58)	0.28 (0.06, 1.22)

*OR, odds ratio; CI, confidence interval*.

*Bolded values are statistically significant (p < 0.05)*.

### Concern Over Virus Exposure and Mitigation Behaviors

**Figure 3:**
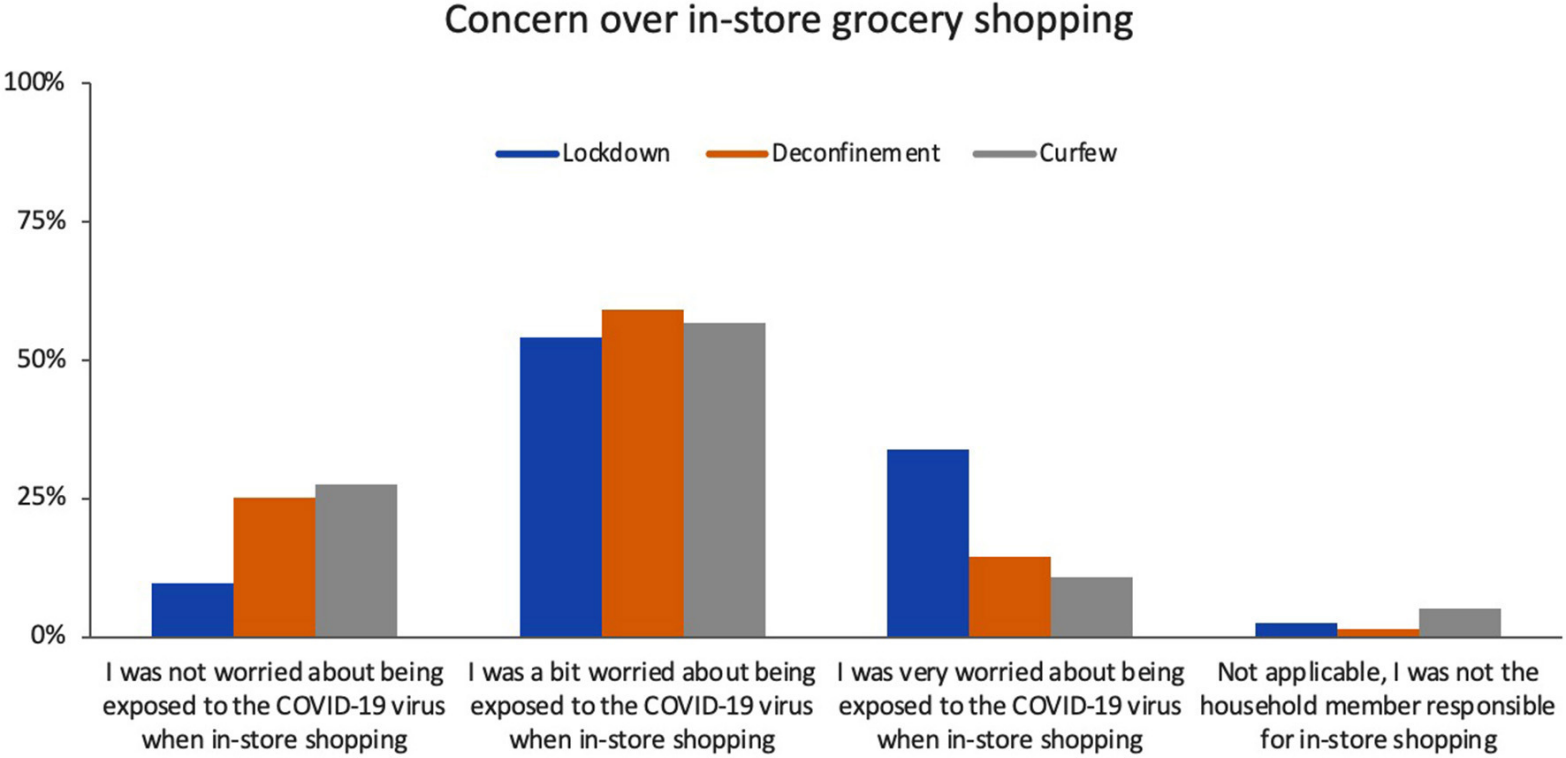
Concerns over in-store grocery shopping. “Between *(time period)*, which of the following statements describes how you felt about possible exposure to the COVID-19 virus when in-store grocery shopping?” Wilcoxon signed-rank test: Lockdown vs. Deconfinement *p* < 0.001, Deconfinement vs. Curfew *p* = 0.239.

**Figure 4:**
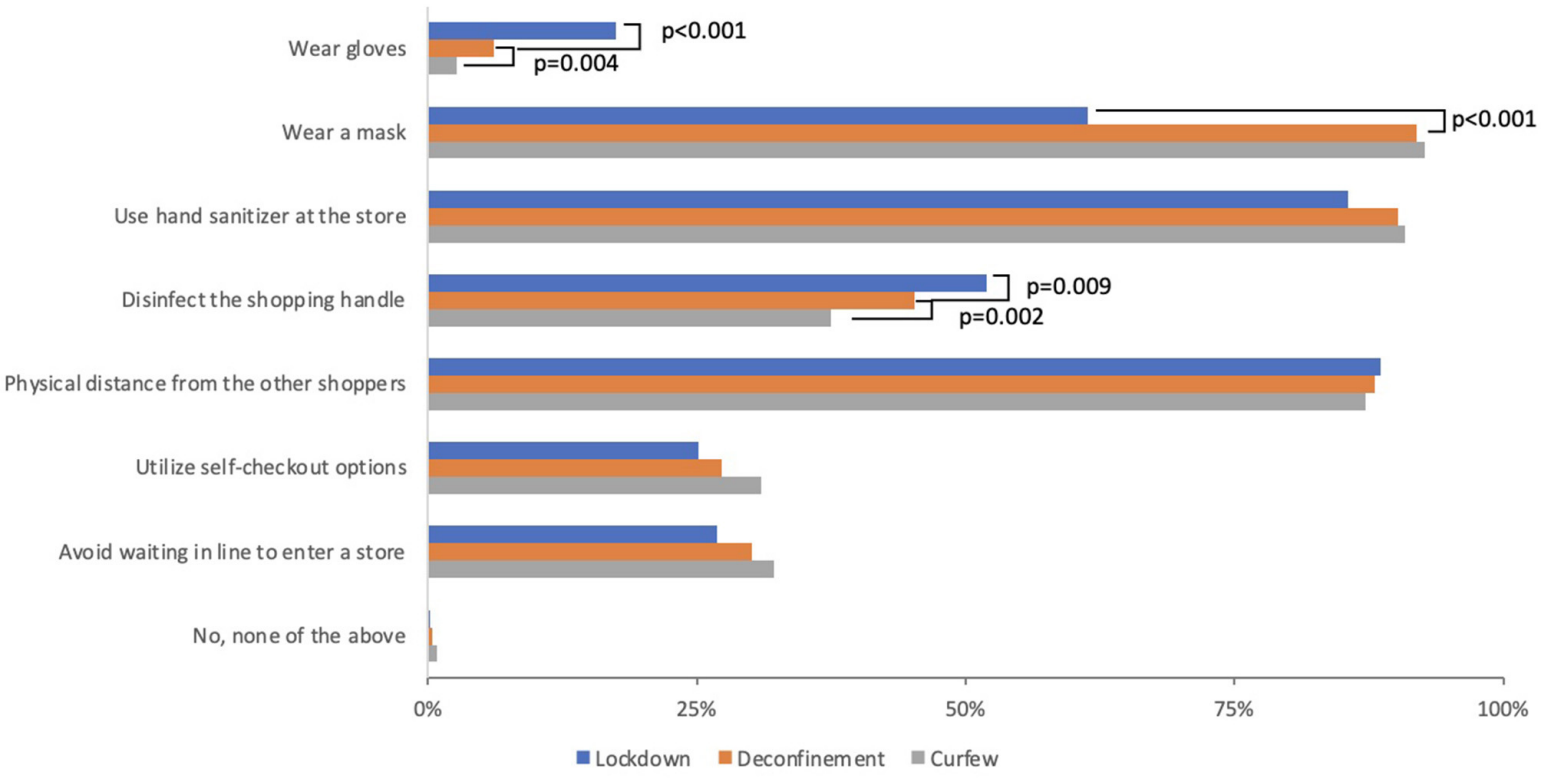
In-store virus exposure mitigation behaviors. “When shopping for groceries in a store did you... (Check boxes if YES and select all that apply).” McNemar test comparing Lockdown (blue) to Deconfinement (orange), and Deconfinement (orange) to Curfew (gray).

During the lockdown, roughly 90% of respondents reported that they were “a bit worried” (54%) or “very worried” (34%) about being exposed to the COVID-19 virus when in-store grocery shopping (Figure 3). Worry significantly differed across time points (Friedman test p < 0.001). Specifically, worry decreased between the lockdown and deconfinement, with roughly 70% of participants reporting being “a bit” (59%) or “very” (14%) worried during deconfinement. This remained stable at the curfew time point. In-store virus mitigation behaviors that significantly changed across time points included wearing gloves, a face mask, and disinfecting the shopping cart handle (Cochran *Q*-test all *p* < 0.001), (Figure 4). Wearing a face mask significantly increased from 61 to 92% between lockdown and deconfinement (face masks became mandatory in indoor public settings in July 2020), thereafter remaining stable. Reports of wearing gloves while shopping and disinfecting the shopping cart handle significantly decreased between the lockdown and the follow-up points (from 18 to 3%, and 52 to 38%, respectively). The most common selected strategies of using hand sanitizer in store and attempting to keep a physical distance from other shoppers remained stable across time points (~90% each). Utilizing self check-out and avoiding wait lines to enter a store also remained stable over time, but were reported by a smaller proportion of respondents (~30%).

**Figure 5:**
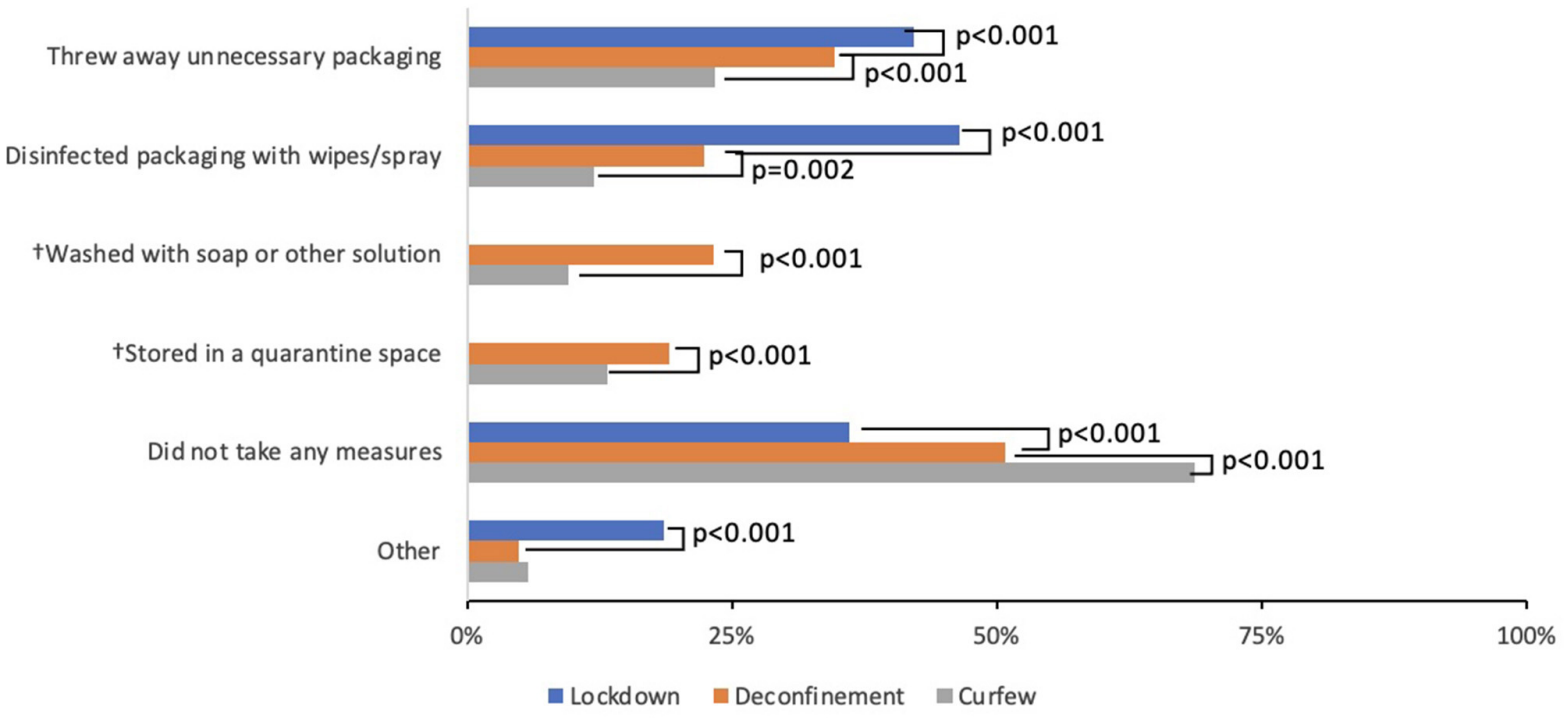
Handling of groceries after purchase. “Between *(time period)* did anyone in your household take measures to disinfect product packaging after getting your groceries? (*Select all that apply)”* McNemar test comparing Lockdown (blue) to Deconfinement (orange), and Deconfinement (orange) to Curfew (gray). †These options were identified as common themes from baseline free text responses to “Other (please specify)”, thus only deconfinement to curfew data are available.

During the lockdown, 46% of respondents reported that they “disinfected packaging with wipes/spray” and 42% “threw away unnecessary packaging” (Figure 5). Analysis of free text responses at this time point revealed two additional measures: washing produce (fresh fruits and vegetables) with soap, vinegar, or diluted bleach and leaving non-perishable items in a “quarantine space” for hours to days before using. As a result, these behaviors were added as response options to the subsequent surveys. The prevalence of all grocery handling strategies significantly decreased after the lockdown and deconfinement periods to ultimately only 12 and 8% of respondents reporting disinfecting packaging and washing packaging with soap, respectively, at the curfew time point (Cochran *Q*-test all *p* < 0.001). Reports of discarding unnecessary packaging and leaving items in a quarantine space were reported by 23 and 13% of respondents, respectively, at the curfew time point.

### Methods of Meal Preparation

Household mode of meal preparation was assessed before (2019), during and after the lockdown (Table 3). A significant increase in the frequency of cooking meals at home was reported during the lockdown compared to 2019. However, the frequency of cooking meals significantly decreased between the lockdown and deconfinement and then remained stable until the curfew period. The frequency of ordering prepared food significantly increased between deconfinement and the curfew period, with a greater proportion of respondents reporting ordering take-out food once per week or more often. The frequency of reporting eating at a sit-down restaurant had significantly decreased between 2019 and deconfinement (the only time point during this study when in-restaurant dining was permitted with capacity limits and distancing requirements).

**Table 3:** Changes in methods of meal preparation before and during COVID-19.

**On average how often did your household..**.	**2019**	**Lockdown**	**Deconfinement**	**Curfew**	***p-*value**	***p*-value**	***p*-value**
**Cook meals at home?** ^†^					**2019 vs. Lockdown**	**Lockdown vs. Deconfinement**	**Deconfinement vs. Curfew**
Daily or more	312 (64%)	424 (87%)	364 (76%)	348 (73%)	<0.001	<0.001	0.580
4–6 times per week	140 (28%)	51 (10%)	83 (17%)	105 (22%)			
2–3 times per week	28 (6%)	10 (2%)	21 (4%)	18 (4%)			
Once per week	8 (2%)	3 (<1%)	4 (<1%)	3 (<1%)			
1–3 times per month	2 (<1%)	0	1 (<1%)	2 (<1%)			
< Once per month/Never	1 (<1%)	0	2 (<1%)	1 (<1%)			
Total	491	488	475	477			
**Order prepared food (take-out or delivery)?** ^†^					**2019 vs. Lockdown**	**Lockdown vs. Deconfinement**	**Deconfinement vs. Curfew**
Daily or more	0	2 (<1%)	1 (<1%)	1 (<1%)	0.027	0.017	<0.001
4–6 times per week	4 (1%)	1 (<1%)	1 (<1%)	3 (<1%)			
2–3 times per week	30 (6%)	18 (4%)	24 (5%)	33 (7%)			
Once per week	83 (17%)	101 (21%)	89 (18%)	128 (26%)			
1–3 times per month	169 (35%)	114 (23%)	163 (33%)	156 (32%)			
< Once per month/Never	203 (42%)	252 (52%)	197 (40%)	162 (34%)			
Total	489	488	475	483			
**Go out to eat at a sit-down restaurant?** ^†^					**2019 vs. Deconfinement**		
Daily or more	4 (1%)	N/A^*^	0	N/A^*^	<0.001		
4–6 times per week	13 (3%)		0				
2–3 times per week	55 (11%)		3 (<1%)				
Once per week	97 (20%)		19 (4%)				
1–3 times per month	190 (39%)		93 (20%)				
< Once per month/Never	128 (26%)		360 (75%)				
Total	489		475				

† *Percentages may not total to 100% due to rounding*.

* *In-restaurant dining was not permitted at these time points and so was not assessed on these surveys*.

*p-values obtained from Wilcoxon signed-rank test*.

### Food Access Challenges

During the lockdown period, few respondents indicated that they were unable to obtain enough food products for their household's need. While food access did not appear to be problematic, canned or frozen fruits and vegetables and grain products had the highest reports of not obtaining enough (8 and 6%, respectively). Nevertheless, the proportion of respondents who reported that they were able to purchase enough of the products to completely meet their household's needs significantly increased between the lockdown and curfew period (Supplementary Figure 1). The shift was a result of fewer respondents selecting that they were able to “mostly” meet their needs (8–23%), and more selecting “completely” (55–89%) at the curfew time point. At both time points, the top selected factor for not obtaining enough was that products were not available in stores, but the proportion of respondents who selected this was markedly higher during the lockdown period (Supplementary Table 6).

As compared to 2019, during the lockdown period, a 17% decrease was observed in respondents reporting that their household had enough of the kinds of foods they wanted to eat (Table 4). This was a result of more participants reporting that although they had enough to eat, it was not always the kinds of foods that were wanted. This changed over time, with significantly greater participants reporting always having enough of the kinds of food they wanted at each time point after the lockdown period, ultimately exhibiting a proportion at the curfew period that was very similar to that of 2019.

**Table 4:** Changes in household food situation.

**Which of the following statements best describes the food eaten in your household during the period of** ***(time period)*****?**^**†**^					
	**2019**	**Lockdown**	**Deconfinement**	**Curfew**	***p*** **-value**
You and other household members always had enough of the kinds of foods you wanted to eat.	455 (93%)	374 (76%)	398 (83%)	439 (90%)	**2019 vs. Lockdown** <0.001
You and other household members had enough to eat, but not always the kinds of food you wanted.	34 (7%)	116 (24%)	77 (16%)	43 (9%)	**Lockdown vs. Deconfinement** 0.004
Sometimes you and other household members did not have enough to eat.	2 (<1%)	1 (<1%)	1 (<1%)	2 (<1%)	
Often you and other household members didn't have enough to eat.	0	0	0	0	**Deconfinement vs. Curfew** <0.001
Total	491	491	476	484	

† *Percentages may not total to 100% due to rounding*.

*p-values obtained from Wilcoxon signed-rank test*.

Between 10 and 15% of respondents reported that they skipped meals or reduced their food intake across the study time points (Supplementary Table 7). Reasons related to food rationing behavior were highest during the lockdown period (up to 25%), but significantly decreased over time (to 14%). While some participants, particularly during the lockdown, reported reducing food intake because food did not last in between grocery trips, assessment of free text responses revealed that this was linked to efforts to reduce the frequency of grocery shopping rather than due to income-related food challenges. Indeed, assessment of the free text responses revealed more common explanations for reducing food intake across the study time points, which were health consciousness considerations (e.g., food intake reduced due to reduced physical activity), inconvenience around food preparation (e.g., irregular schedule, unwillingness to cook), and stress/anxiety/mental health affecting appetite. Similarly, the food insecurity module items included on deconfinement and curfew surveys revealed very low reports of income-related challenges with food and this pattern was stable between the time points (Supplementary Table 7).

During the lockdown period, 11% of respondents indicated that a household member needed to self-isolate for 14 days (Table 5). This significantly changed over time, decreasing to 5% at deconfinement and then increasing to 10% at the curfew time point (Cochran *Q*-test, *p* = 0.002), aligning with the trajectory of COVID-19 prevalence in the province over the time points. The remaining results were examined only descriptively, given the limited sample sizes. Among respondents who reported a household experience with self-isolation, ~40% indicated that the isolation period impacted their household's ability to shop for food during the lockdown and deconfinement periods, which decreased to 25% at the curfew time point. The most frequently reported ways of accessing food during the 14 days of self-isolation were through a household member that did not need to self-isolate, a family/friend who did not live within the household, and a delivery service. Few respondents reported use of community volunteers for groceries or going out to buy food because they live alone (≤ 5%). Assessment of free text responses identified use of food reserves at home as an additional common method.

**Table 5:** Food access during 14-day self-isolation.

	**Lockdown**	**Deconfinement**	**Curfew**
**Between** ***(time period)*****, did any member of your household need to self-isolate or quarantine for 14 days due to COVID-19?**^†^			
Yes	55 (11%)	24 (5%)	48 (10%)
No	436 (89%)	452 (95%)	437 (90%)
Total	491	476	485
Did the 14-day self-isolation/quarantine impact the ability of your household to shop for food?^*^			
Yes	22 (40%)	10 (42%)	12 (25%)
No	33 (60%)	14 (58%)	36 (75%)
Total Respondents^*^	55	24	48
How did your household shop for food during the 14-day period of required self-isolation/quarantine?^*^			
I went out to buy food because I live alone	3 (5%)	0	0
Relied on a household member that did not need to self-isolate	18 (31%)	11 (42%)	25 (51%)
Relied on a family/friend who did not live within our household	20 (34%)	8 (31%)	9 (18%)
Relied on a delivery service	13 (22%)	6 (23%)	10 (20%)
Relied on volunteer grocery shoppers in the community	2 (3%)	1 (4%)	1 (2%)
Other (please specify)	13 (22%)	6 (23%)	10 (20%)
Total Respondents^*^	55	24	48

† *Percentages may not total to 100% due to rounding. Cochran's Q-test comparing responses across all three time points: p = 0.002*.

* *Follow-up question (denominator is the number of respondents who answered “Yes” to the first question). Percentages may total to more than 100% due to the possibility of selecting more than one answer*.

## Discussion

Our findings indicate that the sample of Quebec residents who responded to our three surveys organized themselves around purchasing food in a manner that adhered to government directives for physical distancing depending on the status of the public health situation. In-store grocery shopping frequency was lowest during the lockdown period and mostly done by one member of the household, but, as we had hypothesized, frequency increased after this period to resemble the 2019 pre-pandemic Canadian estimate of 1.3 grocery trips per week (17), and reports of more than one household member going grocery shopping increased after the lockdown. In addition, cooking meals at home significantly increased during the lockdown compared to the report for 2019, which also has been reported internationally in Eastern Europe and the Middle East (18, 19). In line with these observations, the number of daily meals consumed was reported to be higher during lockdown periods compared to before the pandemic in an international online survey study with respondents from Europe, North-Africa, Western Asia and the Americas (20). Frequency of cooking meals at home significantly decreased after the lockdown among our sample, while ordering prepared food significantly increased by our final time point. Together, these observations indicate that our sample of survey respondents complied with the directive to limit outings at the time that it was strictly enforced, and likely also reflects our observation of reduced concern over in-store grocery shopping and exposure through food as the pandemic went on. Indeed, while 11% of respondents reported that they did not go in-store grocery shopping during the lockdown, this decreased by roughly half at the follow-up assessments. In line with this, while many respondents reported disinfecting food product packaging and taking other measures to avoid exposure through grocery products during the lockdown (such as leaving items in a quarantine space), these behaviors decreased over time. The lockdown time point findings were concerning as they paralleled reports of increases in calls to poison control centers due to disinfectant exposures since the beginning of the pandemic, and improper food storage can increase the risk of food-borne illness (21, 22). Consumers likely became less concerned over virus exposure through food and packaging as public health information consistently communicated that the potential to contract the virus through food is very low (23). This highlights the importance of clear and consistent communication of public health information in times of public health emergencies.

Our assessment of no-contact grocery use revealed limitations with the approach during the lockdown period, with nearly 50% of respondents reporting receipt of incomplete grocery orders and ~40% reporting a wait time of 4 days or more. The completeness of and wait time for no-contact grocery methods appeared to have vastly improved by the deconfinement time point and remained stable thereafter. While we can not rule out the possibility that consumers modified their no-contact grocery orders based on the experience during the lockdown period to increase the likelihood of obtaining a complete order, incomplete orders at the beginning of the pandemic may have been a result of lack of retailer preparedness for the sudden higher demand for no-contact methods. Retailers may also have placed limits on the amount of products that they were willing to provide for no-contact orders vs. for patrons shopping in stores, or from actual shortages of some products (24, 25). Although prevalence of no-contact grocery use decreased after the lockdown among our study sample, food retailers likely also adapted to the demand for no-contact orders, which are anticipated to continue to grow in popularity (26).

Contrary to our hypothesis, sociodemographic characteristics of regular users of no-contact grocery methods were not stable over time. The most consistent predictor of regular use of no-contact grocery method was the usual mode of transportation for grocery shopping (public transit, walking or cycling). It is possible that these individuals may have been more likely to have regularly used no-contact grocery methods, particularly home delivery, prior to the pandemic due to preference and issues around transportation. On the other hand, it is also conceivable that pandemic impacts on public transit (reduced hours or concerns over virus exposure) played a role in this observation. Being 60 years of age or older, a risk factor for severe illness from COVID-19 (16), was a significant predictor of regular no-contact grocery use during the lockdown period, suggesting that personal risk perception may have influenced use of no-contact grocery at this time point. Pre-pandemic consumer research indicated that online grocery methods were more popular among younger individuals (26); however, the observation of older individuals utilizing no-contact grocery during the pandemic has been reported both locally in the Quebec context (27), and abroad, such as in China where the elderly population embraced mobile apps for grocery ordering during the first wave lockdown (28). Income was only a significant predictor of regular no-contact grocery use during the lockdown period when use of no-contact grocery methods was highest overall. Therefore, use of no-contact grocery methods during the lockdown was partly driven by higher income households, which is not unexpected given that these methods typically involve an added cost (or minimum purchase) for preparation/delivery of the order. Our previous lockdown assessment of these characteristics also considered regional COVID-19 prevalence (low, medium, high) and found significantly lower likelihood of regular use of no-contact grocery methods among the region with low COVID-19 prevalence (29). A similar result was reported in a framed choice experiment conducted among a sample of US consumers that manipulated COVID-19 case trend according to three scenarios (increasing cases, decreasing cases, or constant) (5). Our results indicate that food retailers may benefit from considering the regional situation and sociodemographic profile around their location when evaluating/refining their no-contact grocery methods, particularly the common modes of transportation.

Food access challenges and indicators of food insecurity were very low among our sample, reflecting the good socioeconomic status of our sample of respondents. In fact, among participants who reported that they skipped meals/reduced their food intake, the reasons were more often related to health consciousness or stress rather than finances. During the lockdown period, we observed an interesting pattern that households appeared to be more rigid with their home food supply compared to pre-pandemic, which noticeably impacted reported *food variety*, but not quantity. Reports of having both enough quantity and variety (kinds) of desired foods increased after the lockdown, ultimately returning to pre-pandemic level. This observation is likely explained by the noticeable reduction in grocery shopping frequency during the lockdown, suggesting that households were making do with food reserves within the home and making fewer trips to obtain desired ingredients/products. Closures of local food suppliers and food shortages during the pandemic have been linked to increased vulnerability to food insecurity in developed countries (30–32), but consistent evidence has demonstrated that the pandemic has caused greater burden among lower socio-economic status groups that are more vulnerable to food and nutrition challenges (30, 33–36). Lockdowns have had the most severe consequences in poor countries as they resulted in complete loss of income for many daily wage workers, representing most of the labor force in low-income countries (37). Government supports including unemployment benefits, postponement of rent and utility payments, financial support for small businesses (including farmers and restaurants), and free food provision are global strategies that have assisted in the food crisis experienced by low socioeconomic status groups throughout the pandemic (37). For example, India provided free weekly rations of rice, pulses, spices, and cooking oil to low-income households during the first wave's lockdown period (38). Humanitarian organizations also provided relief, as evidenced by the WFP and UNICEF provision of rations, vouchers or cash transfers to children in 68 countries due to the closure of schools that had provided nutritious meals to students (39). While these supports should remain in place as necessary, further attention to enhancing the resilience of food systems is also warranted to support the health of people, the environment, and economies. Indeed, supporting local farmers, urban agriculture, and home gardening have been identified as important strategies to help combat food crises that have arisen during the pandemic (40, 41).

We examined food access during self-isolation and our observations indicate that many individuals who experienced self-isolation reported that it impacted the ability to shop for food. While respondents mostly reported obtaining food by relying on individuals (within or outside of their household) who did not need to self-isolate, many also communicated that they relied on their own supply of food reserves in the home during this period. However, households that do not maintain an abundant supply of food (or those that have limited financial or storage capacity) may experience challenges with food sufficiency during a 14-day period of self-isolation, which could impact compliance. Although our observations must be interpreted with caution given our limited sample size of respondents who reported an experience with self-isolation, it may be prudent for public health messages to continue providing consumers with information on proper ways of keeping an adequate food supply at home to maintain a level of preparedness in the event of any future public health emergency. Income support for lower socioeconomic groups should also be available to support sufficient food access (42, 43), and future work should evaluate availability and reliability of no-contact grocery among disadvantaged groups. These strategies will assist both with ensuring that households are prepared in the event of requirements for isolation/quarantine, and with prevention of panic buying occurrences. Indeed, consumer sales data in Canada provide strong evidence of panic buying at the start of the pandemic (44). A surge in sales of non-perishable food items was observed, which aligns with our observation that canned/frozen produce and grain products were the most common products that respondents reported they were not able to obtain enough during the lockdown period. Therefore, while challenges with food access were not prevalent among our sample, our results support anecdotal reports of shortages with certain food products (e.g., non-perishable items) in grocery retailers early in the pandemic, which may have been the result of consumer panic buying.

Despite the strengths of this investigation's provincial coverage and collection of data during the multiple time points over the pandemic, several limitations are worth discussing. First, our sample was comprised of a large proportion of females and high-income bracket households from mostly large urban regions. However, our survey required the respondent to be the individual who was primarily responsible for grocery shopping. The large proportion of female respondents may reflect the observation that women are more likely to take on responsibilities for household food budgeting, purchasing and preparation within households (45). Indeed, women may be more knowledgeable about the household food situation, justifying their suitability as the respondent for household food surveys (46). The sociodemographic profile of our sample may reflect the online recruitment methods that were predominantly used, which were necessary at the time due to the public health restrictions in place. Nevertheless, online methods of recruitment are increasingly recognized for their efficiency and effectiveness and have been increasingly used over the course of the pandemic (47, 48). We did not collect information on certain demographic variables that are linked to challenges with food access (e.g. being a member of an ethnic minority group or Indigenous community), so we were not able to evaluate outcomes with these considerations (49). Despite this, it is unlikely that our sample was representative of such vulnerable groups. Responses were self-reported, which is subject to potential biases and measurement error, and items that required recollection of 2019 may be subject to recall bias. The voluntary option to respond to all survey questions may have resulted in some response bias being present in results, though response rates for each survey question were high. We did not correct for multiple statistical testing due to the exploratory nature of several comparisons given the unprecedented experience of the pandemic. Finally, to our knowledge, no previous work has evaluated characteristics of no-contact grocery users during the pandemic or food access methods during self-isolation. Thus, we are not able to compare our findings for these outcomes with international data.

## Conclusions

Our findings reflect longitudinal patterns of food procurement and related outcomes spanning 1 year among a sample of Quebec households during different periods of the COVID-19 pandemic. In general, concerns of virus exposure in grocery stores and from food packaging were highest during the lockdown period. Several North American and international sources corroborate our observations of decreased in-store grocery shopping, increased use of no-contact grocery methods, and increased cooking at home during the beginning of the pandemic. Lockdown restrictions and concern of in-store grocery shopping appear to be important contributors to those patterns. Overall, our observations support the following recommendations and suggestions for future research: (1) Opportunities exist for continued public health communication regarding food procurement strategies in the event of a future public health emergency as well as messages for appropriate food handling and use of chemical disinfectants; (2) Food retailers and public health agencies may wish to monitor regional availability and reliability of no-contact grocery methods to ensure equitable access and reliable service, especially in times of need; (3) Continued research into food access challenges among vulnerable groups and identification of effective government and local supports; and (4) Global investigations into food procurement activities during the post-pandemic period will be needed to identify and understand lasting impacts on consumer food procurement patterns, particularly pertaining to online food environments.

## Data Availability Statement

The raw data supporting the conclusions of this article will be made available by the authors, without undue reservation.

## Ethics Statement

The studies involving human participants were reviewed and approved by McGill University Faculty of Agricultural and Environmental Sciences Research Ethics Board. The patients/participants provided their written informed consent to participate in this study.

## Author Contributions

DN designed the study, performed statistical analysis, and wrote the first draft of the manuscript. KL, IK, HH, and MT assisted with data collection and statistical analysis. P-GD, LA, CP, and LD provided guidance on the study design and critically revised the manuscript. All authors have read and approved the final manuscript.

## Funding

This study was funded by a McGill Social Sciences and Humanities Research Council (SSHRC) Institutional Grant and was one of the critical research programs being supported by the McGill Interdisciplinary Initiative in Infection and Immunity (MI4) with seed funding from the McGill University Health Centre (MUHC) Foundation (253096).

## Conflict of Interest

The authors declare that the research was conducted in the absence of any commercial or financial relationships that could be construed as a potential conflict of interest.

## Publisher's Note

All claims expressed in this article are solely those of the authors and do not necessarily represent those of their affiliated organizations, or those of the publisher, the editors and the reviewers. Any product that may be evaluated in this article, or claim that may be made by its manufacturer, is not guaranteed or endorsed by the publisher.

## Abbreviations

COVID-19: coronavirus disease 2019.
