# Supplementary material for: Longitudinal Patterns of Food Procurement Over the Course of the COVID-19 Pandemic: Findings From a Canadian Online Household Survey
Source: Front Public Health. 2022 Jan 20;9:752204. doi: 10.3389/fpubh.2021.752204 (PMC8810501; doi:10.3389/fpubh.2021.752204)
Supplement: Supplementary file 1 [file Data_Sheet_1.docx]

**Table S1 Baseline (Lockdown) Survey Demographic Items**

| **Item** | **Response Options** |
| --- | --- |
| Which category includes your age? | - 18-29 - 30-39 - 40-49 - 50-59 - 60-69 - 70-79 - 80 and older |
| What is your gender?  *Gender refers to current gender which may be different from sex assigned at birth and may be different from what is indicated on legal documents.* | - Male - Female - Prefer to specify (enter) |
| To determine which geographic region you live in, please provide your postal code. | Open response |
| Rate how concerned you are about the COVID-19 pandemic. | - Not at all concerned - Slightly concerned - Moderately concerned - Very concerned - Extremely concerned |
| What is your best estimate of the total household income received by all household members, from all sources, before taxes and deductions, in the past 12 months? | - Less than $20,000 - $20,000 or more, but less than $50,000 - $50,000 or more, but less than $100,000 - $100,000 or more, but less than $150,000 - $150,000 or more |
| What was your marital status between the dates of March 13, 2020 and May 4, 2020? | - Single (never legally married) - Common-law relationship - Separated (living together during COVID-19) - Separated (living apart during COVID-19) - Married - Divorced - Widowed |
| Including yourself, how many individuals lived in your household at the start of the COVID-19 pandemic in Quebec (March 13, 2020) | Open response |
| What is your primary mode of transportation for grocery shopping? | - Car - Public transit - Walking - Cycling - Other (please specify) |

**Table S2 Longitudinal Survey Items**

| 1. **Item** | **Response Options** |
| --- | --- |
| Grocery shopping frequency and methods, and methods of meal preparation | |
| 1. 1. Since *(time period)*, which of the statements below best describes your household's approach to in-store grocery shopping? | - I was the only person responsible for in-store grocery shopping during that time period. - Another household member (not me) was responsible for in-store grocery shopping during that time period. - More than one household member went grocery shopping over that time period. - Not applicable, I/we did not grocery shop in-store during that time period (i.e. used delivery or pick-up methods only). |
|  |  |
|  |  |
|  |  |
| 2. Overall, between *(time period)* how often did you/your household delegate physically go into a store to shop for groceries? | - Daily or more - 4-6 times per week - 2-3 times per week - Once per week - 1-3 times per month - Less than once per month - Never |
| 1. 3. Between *(time period)* how often did your household utilize grocery pick-up or home delivery? | - Daily or more - 4-6 times per week - 2-3 times per week - Once per week - 1-3 times per month - Less than once per month/never |
| 1. 4. If you used grocery pick-up or delivery, were all of the products your household ordered included in what you received? | - N/A I did not use this service - Yes everything I/we had ordered - Almost everything I/we had ordered - Some products I/we had ordered were not included - Many products I/we had ordered were not included |
| 1. 5. If you used grocery pick-up or delivery, on average, after placing your order how long did you have to wait to receive your groceries? | - N/A I did not use this service - 1-3 days - 4-7 days - 8-13 days - 2 weeks or more |
| 1. 6. Thinking about the period *(time period),* how often did your household...  - Cook meals at home - Go out to eat at a sit-down restaurant - Ordered prepared food (take-out or delivery) | - Daily or more - 4-6 times per week - 2-3 times per week - Once per week - 1-3 times per month - Less than once per month - Never |
| Concern of virus exposure and mitigation behaviors | |
| 1. 7. Between *(time period),* which of the following statements describes how you felt about possible exposure to the COVID-19 virus when in-store grocery shopping. | - I was not worried about being exposed to the COVID-19 virus when in-store shopping - I was a bit worried about being exposed to the COVID-19 virus when in-store shopping - I was very worried about being exposed to the COVID-19 virus when in-store shopping - Not applicable, I was not the household member responsible for in-store shopping |
| 1. 8. When shopping for groceries in a store did you… (Check boxes if YES and select all that apply) | - Wear gloves - Wear a mask - Use hand sanitizer at the store - Disinfect the shopping cart handle with a disinfectant wipe - Attempt to keep a physical distance from other shoppers - Utilize self-checkout options - Avoid waiting in a line to enter a store - No, none of the above |
| 1. 9. Between *(time period)* did anyone in your household take measures to disinfect product packaging after getting your groceries? Select all that apply. | - Threw away unnecessary packaging - Disinfected packaging with wipes/spray - Washed packaging/products with soap^†^ - Applied other solution to packaging/products (please specify)^†^ - Stored non-perishable items in a quarantine space for a period of time^†^ - No, I/we did not take measures to disinfect product packaging. - Other (please specify)   ^†^Note: These items were only present on the follow-up surveys because they were identified as common responses to Other (please specify) on the baseline survey. |
| Food access challenges and indicators of food insecurity | |
| 10. Between *(time period)*, were you able to obtain enough of the following products to meet your household's needs?   - Dairy products - Dairy alternatives* - Meat or fish products - Meat alternatives* - Fresh fruits and vegetables - Canned or frozen (NOT fresh) fruits and vegetables - Grain products (breads, rice, pasta, oats and other cereals e.g. bran, barley, bulgur)   **Note: Dairy alternatives include fortified soy and other plant-based beverages and yogurt products that may or may not be fortified (e.g., almond, cashew, rice, oat, hemp, or coconut beverages/yogurt products). Meat alternatives include eggs, beans, lentils, peas, chickpeas, nuts, seeds, tofu, and tempeh.* | - Yes, completely - Yes, mostly - No - N/A I do not consume this food product |
| 11. If you weren't able to obtain enough food products, which if any, of the factors listed below were relevant? (Select all that apply) | - I was able to obtain enough of these food products - Not available in store - Could not afford the products - Not included in grocery delivery/pick-up - I live too far from a store that sells these products |
| 1. 12. Which of the following statements best describes the food eaten in your household during the period of *(time period)*. | - You and other household members always had enough of the kinds of foods you wanted to eat. - You and other household members had enough to eat, but not always the kinds of food you wanted. - Sometimes you and other household members did not have enough to eat. - Often you and other household members didn't have enough to eat. |
| 13a. Did anyone in your household skip meals or reduce their food intake between *(time period)*? | - Yes - No - I don’t know |
| 13b. (If above Yes) Were any of the factors listed below reasons for skipping meals or reducing food intake? (Select all that apply.) | - Food did not last in between grocery shopping (from either in-store shopping, delivery, or pick-up) - Could not afford to buy more food - Saving food for children in the household - Saving food for elderly members in the household - Other (please specify) |
|  |  |
| 14. Please select the response that best applies to the following statements: You and other members of your household worried that food would run out before you got money to buy more. Was that often true, sometimes true, or never true in the past 6 months? | - Often true - Sometimes true - Never true - Don’t know |
| 15. You and other members of your household couldn't afford to eat balanced meals. In the past 6 months was that often true, sometimes true, or never true? | - Often true - Sometimes true - Never true - Don’t know |
| Food access during 14-day self-isolation | |
| 16a. Between *(time period)*, did any member of your household need to self-isolate or quarantine for 14 days due to COVID-19? (*Self-isolation and quarantine go above and beyond social distancing and physical distancing. Required self-isolation/quarantine may have occurred due to a positive test result for COVID-19, close contact with a confirmed case, or return to Canada from travel abroad.)* | - Yes - No - Prefer not to answer |
| 16b. (If above Yes) Did the 14-day self-isolation/quarantine impact the ability of your household to shop for food? | - Yes - No |
| 16c. How did your household shop for food during the 14-day period of required self-isolation/quarantine? (Select all that apply.) | - I went out to buy food myself because I live alone - Relied on a household member that did not need to self-isolate - Relied on a family/friend who did not live within our household - Relied on a delivery service - Relied on voluntary grocery shoppers in the community - Other (please specify) |

**Table S3 Addition/removal and reference period of repeated items on longitudinal surveys**

| **Survey** | **Survey Items*** | **Specified reference period** |
| --- | --- | --- |
| Baseline (Lockdown) | 6, 13 only^†^ | Year before COVID-19 (January 2019 – December 2019) |
| Baseline (Lockdown) | All items except 14, 15^‡^ | March 13, 2020 – May 4, 2020 (lockdown period) |
| Follow-up 1 (Deconfinement) | All items except 10, 11^$^ | June 5, 2020 – present (time since baseline survey closure to time of follow-up 1 survey completion) |
| Follow-up 2 (Curfew) | All items | January 9, 2021 – present (start of curfew period to time of follow-up 2 survey completion) |

*Refer to question numbers indicated in Table S2.

^†^Items 6 and 13 were the only questions that queried respondents about the year before COVID-19, and thus were only necessary to be included on the Lockdown survey.

^‡^Items 14 and 15 were added to the Deconfinement and Curfew surveys to better capture income-related food insecurity, because of initial analysis of baseline responses to food security questions.

^$^Items 10 and 11 were removed from the Deconfinement survey, because of the lifting of public health restrictions by that time point. They were re-added to the Curfew survey because restrictions had tightened again by that time point.

**Table S4 Respondent regions of residence**

| **Administrative Health Region** | **n (%)** |
| --- | --- |
| Bas-Saint-Laurent | 7 (1%) |
| Saguenay–Lac-Saint-Jean | 11 (2%) |
| Capitale-Nationale (Québec City) | 37 (8%) |
| Mauricie-et-Centre-du-Québec | 10 (2%) |
| Estrie | 28 (6%) |
| Montréal | 241 (49%) |
| Outaouais | 16 (3%) |
| Abitibi-Témiscamingue | 1 (<1%) |
| Côte-Nord | 1 (<1%) |
| Nord-du-Québec | 0 |
| Gaspésie–Îles-de-la-Madeleine | 3 (<1%) |
| Chaudière-Appalaches | 11 (2%) |
| Laval | 14 (3%) |
| Lanaudière | 18 (4%) |
| Laurentides | 21 (4%) |
| Montérégie | 72 (15%) |
| Nunavik | 0 |
| Terres-Cries-de-la-Baie-James | 0 |

**Table S5 Household member responsible for in-store grocery shopping**

| **Which of the statements below best describes your household's approach to in-store grocery shopping**^†^ | **Lockdown** | **Deconfinement** | **Curfew** |
| --- | --- | --- | --- |
| I was the only person responsible for in-store grocery shopping | 281 (57%) | 258 (54%) | 261 (53%) |
| Another household member (not me) was responsible for in-store grocery shopping | 41 (8%) | 28 (6%) | 23 (5%) |
| More than one household member went grocery shopping | 110 (22%) | 166 (35%) | 175 (36%) |
| Not applicable, I/we did not grocery shop in-store | 58 (11%) | 28 (6%) | 31 (6%) |
| Total | 490 | 480 | 490 |

^†^Percentages may not total to 100% due to rounding.

**Table S6 Reasons for challenges with food access over the course of the pandemic**

| **Food Product** | **Time Point*** | **I was able to obtain enough of these food products** | **Not available in store** | **Could not afford the products** | **Not included in grocery delivery/pick-up** | **I live too far from a store that sells these products** |
| --- | --- | --- | --- | --- | --- | --- |
| Dairy | Lockdown (n=477) | 386 (81%) | 60 (12%) | 2 (<1%) | 18 (4%) | 0 |
|  | Curfew (n=473) | 390 (82%) | 18 (4%) | 2 (<1%) | 4 (1%) | 0 |
| Dairy Alternatives | Lockdown (n=268) | 207 (77%) | 37 (14%) | 4 (2%) | 10 (4%) | 2 (1%) |
|  | Curfew (n=307) | 247 (80%) | 22 (7%) | 2 (<1%) | 4 (1%) | 2 (<1%) |
| Meat and Fish | Lockdown (n=458) | 308 (67%) | 101 (22%) | 15 (3%) | 24 (5%) | 0 |
|  | Curfew (n=454) | 359 (79%) | 24 (5%) | 10 (2%) | 4 (1%) | 1 (<1%) |
| Meat Alternatives | Lockdown (n=355) | 231 (65%) | 103 (29%) | 0 | 17 (5%) | 2 (<1%) |
|  | Curfew (n=332) | 267 (80%) | 29 (9%) | 1 (<1%) | 4 (1%) | 2 (<1%) |
| Fresh Fruits & Vegetables | Lockdown (n=486) | 357 (73%) | 84 (17%) | 16 (3%) | 27 (6%) | 2 (<1%) |
|  | Curfew (n=484) | 356 (74%) | 50 (10%) | 8 (2%) | 10 (2%) | 1 (<1%) |
| Canned/Frozen Fruits & Vegetables | Lockdown (n=423) | 239 (56%) | 165 (39%) | 3 (<1%) | 23 (5%) | 0 |
|  | Curfew (n=437) | 339 (78%) | 40 (9%) | 1 (<1%) | 4 (1%) | 0 |
| Grains | Lockdown (n=483) | 292 (60%) | 161 (33%) | 6 (1%) | 27 (6%) | 0 |
|  | Curfew (n=479) | 381 (80%) | 31 (6%) | 1 (<1%) | 2 (<1%) | 1 (<1%) |

Select all that apply question. Percentages may total to more than 100% due to the possibility of selecting more than one answer.

*Respondents who selected “N/A I do not consume this food product” were excluded from analyses.

**Table S7 Changes in indicators of food security**

| **Did anyone in your household skip meals or reduce their food intake between *(time period).***^†^ | | | | | |
| --- | --- | --- | --- | --- | --- |
|  | **Lockdown** | **Deconfinement** | | **Curfew** | **p-value** |
| Yes | 47 (10%) | 70 (15%) | | 58 (12%) | **Overall**  0.013  **Lockdown vs. Deconfinement**  0.002  **Deconfinement vs. Curfew**  0.113 |
| No/I don’t know | 443 (91%) | 407 (85%) | | 426 (88%) |  |
| Total | 490 | 477 | | 484 |  |
| **Were any of the factors listed below reasons for skipping meals or reducing food intake? (Select all that apply)*** | | | | | |
|  | **Lockdown** | **Deconfinement** | | **Curfew** |  |
| Food did not last between grocery trips (either from in-store shopping, grocery pick-up or delivery) | 12 (25%) | 11 (16%) | | 8 (14%) |  |
| Could not afford to buy more food | 2 (4%) | 4 (6%) | | 3 (5%) |  |
| Saving food for children in the household | 5 (11%) | 3 (4%) | | 4 (7%) |  |
| Saving food for elderly household members | 1 (2%) | 1 (1%) | | 0 |  |
| Other (please specify) | 28 (60%) | 53 (75%) | | 44 (76%) |  |
| Total Respondents* | 47 | 70 | | 58 |  |
| **You and other members of your household worried that food would run out before you got money to buy more.**^†^ | | | | | |
|  | **Deconfinement** | | **Curfew** | | **p-value** |
| Often true | 4 (1%) | | 2 (<1%) | | 0.156 |
| Sometimes true | 28 (6%) | | 21 (4%) | |  |
| Never true | 445 (93%) | | 458 (95%) | |  |
| Total | 477 | | 481 | |  |
| **You and other members of your household couldn't afford to eat balanced meals.** | | | | | |
|  | **Deconfinement** | | **Curfew** | | **p-value** |
| Often true | 2 (<1%) | | 3 (<1%) | | 0.070 |
| Sometimes true | 16 (3%) | | 23 (5%) | |  |
| Never true | 457 (96%) | | 458 (95%) | |  |
| Total | 475 | | 484 | |  |

^†^Percentages may not total to 100% due to rounding. P-value for overall result obtained from Friedman’s test.

*Follow-up question (denominator is the number of respondents who answered “Yes” to previous question). Percentages may total to more than 100% due to the possibility of selecting more than one answer.

**Figure S1 Challenges in availability of food products**^†^

“Between *(time period)*, were you able to obtain enough of the following products to meet your household's needs? *Note: Dairy alternatives include fortified soy and other plant-based beverages and yogurt products that may or may not be fortified (e.g., almond, cashew, rice, oat, hemp, or coconut beverages/yogurt products). Meat alternatives include eggs, beans, lentils, peas, chickpeas, nuts, seeds, tofu, and tempeh.”*

^†^Asked only on Lockdown and Curfew surveys.

Friedman test p-value < 0.001 for all food product categories
